# 4-(4-Meth­oxy­phen­yl)naphtho­[2,3-*b*]thio­phene

**DOI:** 10.1107/S1600536812005697

**Published:** 2012-02-29

**Authors:** S. Vasudhevan, G. Puthilibai, R. Joel Karunakaran

**Affiliations:** a Department of Chemistry, Madras Christian College (Autonomous), Chennai 600 059, Tamil Nadu, India; b Department of Chemistry, Sri Sairam Engineering College, Chennai 600 044, Tamil Nadu, India

## Abstract

In the title compound, C_19_H_14_OS, the naphtho­thio­phene moiety is almost planar except for the S atom of the five-membered ring, which is situated 0.047 (6) Å out of the C_4_ plane (with an r.m.s. deviation of fitted atoms = 0.0009 Å). The dihedral angle between the naphtho­thio­phene plane and the attached meth­oxy­phenyl ring is 67.6 (2)°. In the crystal, a C—H⋯π inter­action is observed between a meth­oxy­phenyl C—H group and the outer benzene ring of the naphtho­thio­phene moiety. The five-membered ring of the naphtho­thio­phene moiety is disordered, with the S and opposite non-fused C atom approximately exchanging positions, with a site-occupancy factors of 0.808 (3) and 0.187 (3).

## Related literature

For related thio­phene structures, see: Labat & Halfpenny (2005 ▶); Thenmozhi *et al.* (2008 ▶). For related heterocyclic compounds, see: Jones *et al.* (1984 ▶); Palani *et al.* (2006 ▶). For biological activity of naphtho­thio­phenes, see: Zuse *et al.* (2007 ▶, 2006 ▶); Dallemagne *et al.* (2003 ▶); Misra & Amin (1990 ▶).
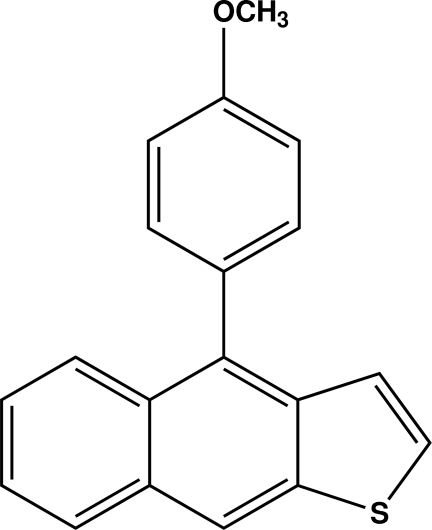


## Experimental

### 

#### Crystal data

C_19_H_14_OS
*M* *_r_* = 290.36
Monoclinic, 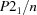

*a* = 9.2961 (5) Å
*b* = 15.9931 (7) Å
*c* = 10.0896 (6) Å
β = 104.580 (3)°
*V* = 1451.75 (13) Å^3^
*Z* = 4
Mo *K*α radiation
μ = 0.22 mm^−1^
*T* = 293 K
0.30 × 0.20 × 0.20 mm

#### Data collection

Bruker Kappa APEXII CCD diffractometer
Absorption correction: multi-scan (*SADABS*; Bruker, 2004 ▶) *T*_min_ = 0.912, *T*_max_ = 0.961
15008 measured reflections
2553 independent reflections
2223 reflections with *I* > 2σ(*I*)
*R*_int_ = 0.024

#### Refinement

*R*[*F* ^2^ > 2σ(*F* ^2^)] = 0.054
*wR*(*F* ^2^) = 0.150
*S* = 1.11
2553 reflections
196 parameters
4 restraints
H-atom parameters constrained
Δρ_max_ = 0.33 e Å^−3^
Δρ_min_ = −0.35 e Å^−3^

### 

Data collection: *APEX2* (Bruker, 2004 ▶); cell refinement: *APEX2* and *SAINT* (Bruker, 2004 ▶); data reduction: *SAINT* and *XPREP* (Bruker, 2004 ▶); program(s) used to solve structure: *SIR92* (Altomare *et al.*, 1993 ▶); program(s) used to refine structure: *SHELXL97* (Sheldrick, 2008 ▶); molecular graphics: *ORTEP-3* (Farrugia, 1997 ▶) and *Mercury* (Macrae *et al.*, 2008 ▶); software used to prepare material for publication: *SHELXL97*.

## Supplementary Material

Crystal structure: contains datablock(s) global, I. DOI: 10.1107/S1600536812005697/im2353sup1.cif

Structure factors: contains datablock(s) I. DOI: 10.1107/S1600536812005697/im2353Isup2.hkl

Supplementary material file. DOI: 10.1107/S1600536812005697/im2353Isup3.cml

Additional supplementary materials:  crystallographic information; 3D view; checkCIF report

## Figures and Tables

**Table 1:** Hydrogen-bond geometry (Å, °) *Cg* is the centroid of the of C5–C10 ring.

*D*—H⋯*A*	*D*—H	H⋯*A*	*D*⋯*A*	*D*—H⋯*A*
C17—H17⋯*Cg*^i^	0.93	2.82	3.621 (3)	145

Symmetry code: (i) 
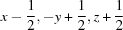
.

**Fig. 1.**
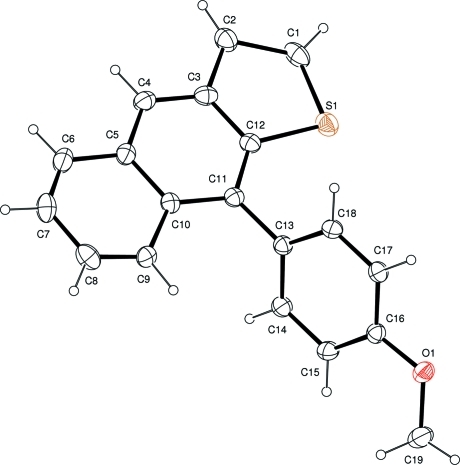
ORTEP plot of the title compound with thermal ellipsoids drawn at the 40% probability level.

**Fig. 2.**
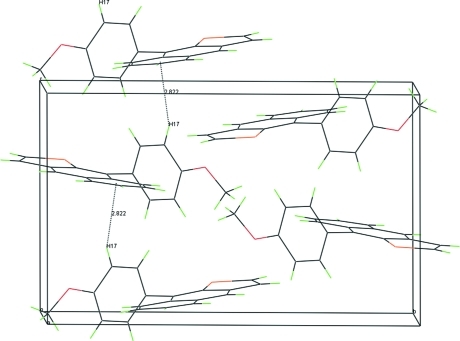
Molecular packing diagram. C—H···π interactions are shown as dotted lines.

## supplementary crystallographic information

### Comment

Biological evaluation of benzo/naphtho thiophene heterocycles has shown them to act as potential antibiological agents. Naphtho[2,3-*b*]thiophene derivatives were found to exhibit antiproliferative activity related to inhibition of tubulin polymerization (Zuse *et al.* 2007, 2006). Five-membered heterocycles fused to cyclopenta[*c*]thiophene are investigated as new antitumor agents (Dallemagne *et al.* 2003). Benzo[*b*]naphtho[2,1-*d*]thiophene derivatives are found as potentially important metabolites (Misra & Amin, 1990).

Fig. 1 shows the *ORTEP* representation of the molecular structure of the title compound with atoms at the 40% probability level. The five membered ring of the naphtho-thiophene moiety is disordered with sulfur (S1) and carbon (C2) approximately exchanging positions. The two portions of the moiety were assigned variable occupancy factors during refinement with sum of their occupancies restrained as 1 resulting in a refined s.o.f. ratio of 81% and 19%. In the naphtho thiophene moiety, S1 of the five membered ring in the major component is observed 0.047 (6) Å out of the (C1–C4) plane (0.058 (7) Å for the minor component). The latter atoms show a r.m.s. deviation of 0.0009 Å or 0.009 Å for the major and minor component, respectively. The dihedral angle between the mean plane of the naphtho-thiophene moiety and the methoxy-phenyl ring is 67.6 (2)°. Fig. 2 shows the packing of molecules in the unit cell. There is a C—H···π interaction between H17 (symmcode: 1.5 - *x*, -0.5 + *y*, 1.5 - *z*) and the phenyl ring (C5–C10). The distance between the H atom and centroid of the phenyl ring is 2.822 Å. Except for this C—H···π interaction, the packing is featureless.

### Experimental

To a solution of (2-(acetoxy(4-methoxyphenyl)methyl)phenyl) (thiophen-2-yl)methylacetate (0.5 g, 1.12 mmol) in anhydrous dichloromethane (20 ml), BF_3_ × Et_2_O (40 mg) was added. The reaction mixture was then stirred at room temperature for 4 h under N_2_ atmosphere. Removal of the solvent followed by column chromatography (n-hexane/ethylacetate 98:2) led to the isolation of the product 4-(4-methoxyphenyl)naphtho[2,3-*b*]thiophene (yield: 56%). The product was recrystallized from chloroform and was obtained as a colourless crystals.

### Refinement

As S1 and C2 are disordered, refinement is carried out with a second component for the thiophene ring with atoms C2', C1', S1' located near S1, C1 and C2, respectively. Disordered pairs of the same sites were restrained to have same thermal parameter. The distance C3—S1' was restrained to be 1.70 Å. Similarily, distances C1—C2' and C2'—C12 were restrained to be around 1.4 Å and 1.52 Å during the refinement. The two components were assigned variable occupancy factors during refinement with the sum of their occupancies restrained as 1 resulting in a refined s.o.f. ratio of 81% and 19%. H atoms were constrained as riding atoms with d(C—H) = 0.93 Å and *U*_iso_(H) = 1.2*U*_eq_(C) for aromatic CH groups and 0.96 Å and *U*_iso_(H) = 1.5*U*_eq_(C) for the methyl group.

### Crystal data

**Table:**

C_19_H_14_OS	*F*(000) = 608
*M**_r_* = 290.36	*D*_x_ = 1.328 Mg m^−^^3^
Monoclinic, *P*2_1_/*n*	Mo *K*α radiation, λ = 0.71073 Å
Hall symbol: -P 2yn	Cell parameters from 4322 reflections
*a* = 9.2961 (5) Å	θ = 2.7–24.5°
*b* = 15.9931 (7) Å	µ = 0.22 mm^−^^1^
*c* = 10.0896 (6) Å	*T* = 293 K
β = 104.580 (3)°	Block, pale yellow
*V* = 1451.75 (13) Å^3^	0.30 × 0.20 × 0.20 mm
*Z* = 4	

### Data collection

**Table:**

Bruker Kappa APEXII CCD diffractometer	2553 independent reflections
Radiation source: fine-focus sealed tube	2223 reflections with *I* > 2σ(*I*)
Graphite monochromator	*R*_int_ = 0.024
ω and φ scans	θ_max_ = 25.0°, θ_min_ = 2.4°
Absorption correction: multi-scan (*SADABS*; Bruker, 2004)	*h* = −11→11
*T*_min_ = 0.912, *T*_max_ = 0.961	*k* = −19→19
15008 measured reflections	*l* = −11→11

### Refinement

**Table:**

Refinement on *F*^2^	Primary atom site location: structure-invariant direct methods
Least-squares matrix: full	Secondary atom site location: difference Fourier map
*R*[*F*^2^ > 2σ(*F*^2^)] = 0.054	Hydrogen site location: inferred from neighbouring sites
*wR*(*F*^2^) = 0.150	H-atom parameters constrained
*S* = 1.11	*w* = 1/[σ^2^(*F*_o_^2^) + (0.0618*P*)^2^ + 1.0365*P*] where *P* = (*F*_o_^2^ + 2*F*_c_^2^)/3
2553 reflections	(Δ/σ)_max_ < 0.001
196 parameters	Δρ_max_ = 0.33 e Å^−^^3^
4 restraints	Δρ_min_ = −0.35 e Å^−^^3^

### Special details

**Table:**

Geometry. All e.s.d.'s (except the e.s.d. in the dihedral angle between two l.s. planes) are estimated using the full covariance matrix. The cell e.s.d.'s are taken into account individually in the estimation of e.s.d.'s in distances, angles and torsion angles; correlations between e.s.d.'s in cell parameters are only used when they are defined by crystal symmetry. An approximate (isotropic) treatment of cell e.s.d.'s is used for estimating e.s.d.'s involving l.s. planes.
Refinement. Refinement of *F*^2^ against ALL reflections. The weighted *R*-factor *wR* and goodness of fit *S* are based on *F*^2^, conventional *R*-factors *R* are based on *F*, with *F* set to zero for negative *F*^2^. The threshold expression of *F*^2^ > σ(*F*^2^) is used only for calculating *R*-factors(gt) *etc*. and is not relevant to the choice of reflections for refinement. *R*-factors based on *F*^2^ are statistically about twice as large as those based on *F*, and *R*- factors based on ALL data will be even larger.

### Fractional atomic coordinates and isotropic or equivalent isotropic displacement parameters (Å^2^)

**Table:**

	*x*	*y*	*z*	*U*_iso_*/*U*_eq_	Occ. (<1)
C1	0.5082 (5)	−0.0475 (3)	0.6960 (6)	0.0644 (14)	0.808 (3)
H1	0.4432	−0.0894	0.7079	0.077*	0.808 (3)
C2	0.6679 (6)	−0.0682 (3)	0.6871 (5)	0.0614 (3)	0.808 (3)
H2	0.7162	−0.1196	0.6943	0.074*	0.808 (3)
S1	0.45408 (12)	0.05754 (8)	0.68296 (10)	0.0614 (3)	0.808 (3)
C1'	0.461 (3)	−0.0280 (15)	0.677 (3)	0.0644 (14)	0.187 (3)
H1'	0.3766	−0.0550	0.6898	0.077*	0.187 (3)
C2'	0.4755 (15)	0.0563 (14)	0.646 (3)	0.0614 (3)	0.187 (3)
H2'	0.3939	0.0917	0.6199	0.074*	0.187 (3)
S1'	0.6213 (5)	−0.0676 (3)	0.6865 (5)	0.0614 (3)	0.187 (3)
C3	0.7222 (3)	0.01533 (15)	0.6642 (3)	0.0490 (6)	
C4	0.8635 (3)	0.02564 (16)	0.6515 (3)	0.0515 (6)	
H4	0.9258	−0.0204	0.6564	0.062*	
C5	0.9148 (3)	0.10577 (17)	0.6308 (2)	0.0461 (6)	
C6	1.0633 (3)	0.1195 (2)	0.6226 (3)	0.0605 (8)	
H6	1.1268	0.0741	0.6279	0.073*	
C7	1.1118 (3)	0.1930 (2)	0.6081 (3)	0.0631 (8)	
H7	1.2106	0.1997	0.6059	0.076*	
C8	1.0209 (3)	0.2633 (2)	0.5954 (3)	0.0630 (8)	
H8	1.0603	0.3153	0.5833	0.076*	
C9	0.8833 (3)	0.25843 (16)	0.6001 (2)	0.0452 (6)	
H9	0.8252	0.3064	0.5910	0.054*	
C10	0.8187 (3)	0.17623 (15)	0.6198 (2)	0.0407 (5)	
C11	0.6717 (3)	0.16559 (15)	0.6331 (2)	0.0409 (5)	
C12	0.6285 (3)	0.08554 (15)	0.6572 (2)	0.0437 (6)	
C13	0.5697 (2)	0.23754 (15)	0.6292 (2)	0.0409 (5)	
C14	0.5147 (3)	0.28478 (16)	0.5126 (3)	0.0484 (6)	
H14	0.5416	0.2712	0.4325	0.058*	
C15	0.4202 (3)	0.35206 (17)	0.5124 (3)	0.0490 (6)	
H15	0.3842	0.3830	0.4328	0.059*	
C16	0.3800 (3)	0.37276 (15)	0.6307 (2)	0.0426 (6)	
C17	0.4321 (3)	0.32557 (16)	0.7474 (3)	0.0461 (6)	
H17	0.4038	0.3387	0.8269	0.055*	
C18	0.5258 (3)	0.25921 (16)	0.7463 (2)	0.0457 (6)	
H18	0.5606	0.2281	0.8259	0.055*	
C19	0.2441 (4)	0.4937 (2)	0.5322 (3)	0.0717 (9)	
H19A	0.3303	0.5160	0.5086	0.107*	
H19B	0.1878	0.5385	0.5576	0.107*	
H19C	0.1838	0.4645	0.4548	0.107*	
O1	0.2893 (2)	0.43755 (11)	0.64381 (19)	0.0548 (5)	

### Atomic displacement parameters (Å^2^)

**Table:**

	*U*^11^	*U*^22^	*U*^33^	*U*^12^	*U*^13^	*U*^23^
C1	0.057 (4)	0.077 (3)	0.056 (3)	−0.031 (2)	0.010 (3)	0.005 (2)
C2	0.0525 (6)	0.0696 (6)	0.0625 (7)	−0.0089 (4)	0.0150 (4)	0.0068 (5)
S1	0.0525 (6)	0.0696 (6)	0.0625 (7)	−0.0089 (4)	0.0150 (4)	0.0068 (5)
C1'	0.057 (4)	0.077 (3)	0.056 (3)	−0.031 (2)	0.010 (3)	0.005 (2)
C2'	0.0525 (6)	0.0696 (6)	0.0625 (7)	−0.0089 (4)	0.0150 (4)	0.0068 (5)
S1'	0.0525 (6)	0.0696 (6)	0.0625 (7)	−0.0089 (4)	0.0150 (4)	0.0068 (5)
C3	0.0631 (16)	0.0428 (14)	0.0391 (13)	0.0014 (12)	0.0091 (11)	0.0000 (10)
C4	0.0606 (16)	0.0466 (14)	0.0472 (14)	0.0126 (12)	0.0137 (12)	0.0023 (12)
C5	0.0438 (13)	0.0570 (15)	0.0371 (13)	0.0057 (11)	0.0094 (10)	−0.0017 (11)
C6	0.0478 (15)	0.080 (2)	0.0544 (17)	0.0155 (15)	0.0140 (12)	0.0006 (15)
C7	0.0409 (14)	0.084 (2)	0.0650 (18)	−0.0075 (14)	0.0153 (13)	−0.0171 (16)
C8	0.074 (2)	0.0624 (18)	0.0568 (17)	−0.0210 (15)	0.0235 (15)	−0.0096 (14)
C9	0.0533 (15)	0.0466 (14)	0.0373 (12)	0.0071 (11)	0.0145 (11)	−0.0074 (10)
C10	0.0422 (12)	0.0470 (13)	0.0331 (11)	−0.0018 (10)	0.0096 (9)	−0.0044 (10)
C11	0.0443 (13)	0.0436 (13)	0.0341 (12)	0.0007 (10)	0.0085 (10)	−0.0003 (10)
C12	0.0447 (13)	0.0463 (14)	0.0378 (12)	−0.0039 (11)	0.0062 (10)	0.0018 (10)
C13	0.0390 (12)	0.0432 (13)	0.0406 (13)	−0.0014 (10)	0.0101 (10)	0.0012 (10)
C14	0.0562 (15)	0.0538 (15)	0.0377 (13)	0.0060 (12)	0.0165 (11)	0.0018 (11)
C15	0.0533 (14)	0.0518 (15)	0.0405 (13)	0.0071 (12)	0.0091 (11)	0.0084 (11)
C16	0.0380 (12)	0.0423 (13)	0.0470 (14)	−0.0008 (10)	0.0100 (10)	−0.0010 (11)
C17	0.0497 (14)	0.0503 (14)	0.0408 (13)	0.0018 (11)	0.0162 (11)	−0.0008 (11)
C18	0.0496 (14)	0.0489 (14)	0.0383 (13)	0.0030 (11)	0.0105 (11)	0.0062 (11)
C19	0.081 (2)	0.0627 (19)	0.073 (2)	0.0271 (16)	0.0225 (17)	0.0152 (16)
O1	0.0584 (11)	0.0520 (11)	0.0562 (11)	0.0144 (9)	0.0185 (9)	0.0041 (9)

### Geometric parameters (Å, º)

**Table:**

C1—C2	1.545 (6)	C8—C9	1.294 (4)
C1—S1	1.749 (5)	C8—H8	0.9300
C1—H1	0.9300	C9—C10	1.479 (3)
C2—C3	1.468 (5)	C9—H9	0.9300
C2—H2	0.9300	C10—C11	1.417 (3)
S1—C12	1.764 (3)	C11—C12	1.381 (3)
C1'—C2'	1.399 (10)	C11—C13	1.486 (3)
C1'—S1'	1.60 (3)	C13—C14	1.384 (3)
C1'—H1'	0.9300	C13—C18	1.387 (3)
C2'—C12	1.474 (10)	C14—C15	1.389 (4)
C2'—H2'	0.9300	C14—H14	0.9300
S1'—C3	1.673 (5)	C15—C16	1.378 (4)
C3—C4	1.362 (4)	C15—H15	0.9300
C3—C12	1.412 (4)	C16—O1	1.363 (3)
C4—C5	1.401 (4)	C16—C17	1.379 (3)
C4—H4	0.9300	C17—C18	1.375 (3)
C5—C6	1.420 (4)	C17—H17	0.9300
C5—C10	1.425 (3)	C18—H18	0.9300
C6—C7	1.281 (4)	C19—O1	1.419 (3)
C6—H6	0.9300	C19—H19A	0.9600
C7—C8	1.392 (4)	C19—H19B	0.9600
C7—H7	0.9300	C19—H19C	0.9600
			
C2—C1—S1	117.7 (4)	C11—C10—C5	119.9 (2)
C2—C1—H1	121.2	C11—C10—C9	123.6 (2)
S1—C1—H1	121.2	C5—C10—C9	116.4 (2)
C3—C2—C1	100.8 (4)	C12—C11—C10	117.3 (2)
C3—C2—H2	129.6	C12—C11—C13	120.7 (2)
C1—C2—H2	129.6	C10—C11—C13	122.0 (2)
C1—S1—C12	89.8 (2)	C11—C12—C3	122.9 (2)
C2'—C1'—S1'	105 (2)	C11—C12—C2'	126.8 (10)
C2'—C1'—H1'	127.7	C3—C12—C2'	108.8 (10)
S1'—C1'—H1'	127.7	C11—C12—S1	125.5 (2)
C1'—C2'—C12	115.9 (19)	C3—C12—S1	111.67 (19)
C1'—C2'—H2'	122.1	C2'—C12—S1	13.0 (10)
C12—C2'—H2'	122.1	C14—C13—C18	117.6 (2)
C1'—S1'—C3	103.0 (9)	C14—C13—C11	122.8 (2)
C4—C3—C12	119.8 (2)	C18—C13—C11	119.6 (2)
C4—C3—C2	120.3 (3)	C13—C14—C15	121.5 (2)
C12—C3—C2	120.0 (3)	C13—C14—H14	119.2
C4—C3—S1'	134.1 (3)	C15—C14—H14	119.2
C12—C3—S1'	106.1 (3)	C16—C15—C14	119.6 (2)
C2—C3—S1'	13.9 (3)	C16—C15—H15	120.2
C3—C4—C5	119.8 (2)	C14—C15—H15	120.2
C3—C4—H4	120.1	O1—C16—C15	125.2 (2)
C5—C4—H4	120.1	O1—C16—C17	115.2 (2)
C4—C5—C6	121.5 (3)	C15—C16—C17	119.6 (2)
C4—C5—C10	120.3 (2)	C18—C17—C16	120.2 (2)
C6—C5—C10	118.2 (3)	C18—C17—H17	119.9
C7—C6—C5	121.6 (3)	C16—C17—H17	119.9
C7—C6—H6	119.2	C17—C18—C13	121.5 (2)
C5—C6—H6	119.2	C17—C18—H18	119.3
C6—C7—C8	121.9 (3)	C13—C18—H18	119.3
C6—C7—H7	119.1	O1—C19—H19A	109.5
C8—C7—H7	119.1	O1—C19—H19B	109.5
C9—C8—C7	122.1 (3)	H19A—C19—H19B	109.5
C9—C8—H8	119.0	O1—C19—H19C	109.5
C7—C8—H8	119.0	H19A—C19—H19C	109.5
C8—C9—C10	119.8 (2)	H19B—C19—H19C	109.5
C8—C9—H9	120.1	C16—O1—C19	118.1 (2)
C10—C9—H9	120.1		
			
S1—C1—C2—C3	−1.9 (5)	C10—C11—C12—S1	177.76 (18)
C2—C1—S1—C12	2.4 (4)	C13—C11—C12—S1	1.0 (3)
S1'—C1'—C2'—C12	−11 (3)	C4—C3—C12—C11	2.2 (4)
C2'—C1'—S1'—C3	4 (2)	C2—C3—C12—C11	−178.7 (3)
C1—C2—C3—C4	179.4 (3)	S1'—C3—C12—C11	−177.1 (3)
C1—C2—C3—C12	0.2 (4)	C4—C3—C12—C2'	168.8 (11)
C1—C2—C3—S1'	−5.9 (12)	C2—C3—C12—C2'	−12.0 (11)
C1'—S1'—C3—C4	−174.8 (13)	S1'—C3—C12—C2'	−10.5 (11)
C1'—S1'—C3—C12	4.3 (13)	C4—C3—C12—S1	−177.7 (2)
C1'—S1'—C3—C2	179 (2)	C2—C3—C12—S1	1.4 (3)
C12—C3—C4—C5	−0.3 (4)	S1'—C3—C12—S1	3.0 (3)
C2—C3—C4—C5	−179.4 (3)	C1'—C2'—C12—C11	−179.4 (19)
S1'—C3—C4—C5	178.8 (3)	C1'—C2'—C12—C3	15 (3)
C3—C4—C5—C6	177.4 (2)	C1'—C2'—C12—S1	−90 (5)
C3—C4—C5—C10	−1.5 (4)	C1—S1—C12—C11	178.0 (3)
C4—C5—C6—C7	−177.8 (3)	C1—S1—C12—C3	−2.1 (3)
C10—C5—C6—C7	1.2 (4)	C1—S1—C12—C2'	78 (4)
C5—C6—C7—C8	−1.9 (5)	C12—C11—C13—C14	−116.0 (3)
C6—C7—C8—C9	1.2 (5)	C10—C11—C13—C14	67.3 (3)
C7—C8—C9—C10	0.2 (4)	C12—C11—C13—C18	63.7 (3)
C4—C5—C10—C11	1.5 (3)	C10—C11—C13—C18	−112.9 (3)
C6—C5—C10—C11	−177.4 (2)	C18—C13—C14—C15	0.7 (4)
C4—C5—C10—C9	179.2 (2)	C11—C13—C14—C15	−179.6 (2)
C6—C5—C10—C9	0.2 (3)	C13—C14—C15—C16	0.1 (4)
C8—C9—C10—C11	176.7 (2)	C14—C15—C16—O1	179.5 (2)
C8—C9—C10—C5	−0.9 (3)	C14—C15—C16—C17	−1.1 (4)
C5—C10—C11—C12	0.3 (3)	O1—C16—C17—C18	−179.3 (2)
C9—C10—C11—C12	−177.2 (2)	C15—C16—C17—C18	1.2 (4)
C5—C10—C11—C13	177.0 (2)	C16—C17—C18—C13	−0.4 (4)
C9—C10—C11—C13	−0.5 (3)	C14—C13—C18—C17	−0.5 (4)
C10—C11—C12—C3	−2.1 (3)	C11—C13—C18—C17	179.7 (2)
C13—C11—C12—C3	−178.9 (2)	C15—C16—O1—C19	−6.8 (4)
C10—C11—C12—C2'	−166.3 (12)	C17—C16—O1—C19	173.7 (3)
C13—C11—C12—C2'	17.0 (13)		

### Hydrogen-bond geometry (Å, º)

**Table:**

*D*—H···*A*	*D*—H	H···*A*	*D*···*A*	*D*—H···*A*
C17—H17···*Cg*^i^	0.93	2.82	3.621 (3)	145

Symmetry code: (i) *x*−1/2, −*y*+1/2, *z*+1/2.
